# Supplementary material for: Impact of delayed response on wearable cognitive assistance
Source: PLoS One. 2021 Mar 23;16(3):e0248690. doi: 10.1371/journal.pone.0248690 (PMC7987160; doi:10.1371/journal.pone.0248690)
Supplement: Attachment — Submitted filename: Response_to_Reviewers.pdf [file pone.0248690.s001.pdf]

February 17, 2021

M. Usman Ashraf, Ph.D
Academic Editor
PLOS ONE

Manuscript PONE-D-20-32471
Response to reviewers

Dear Dr. Usman Ashraf:

We would like to begin by thanking you for giving us the opportunity to submit a revised version of our manuscript *"Impact of delayed response on Wearable Cognitive Assistance"*. We also wish to thank you and the reviewers for the effort you have dedicated to providing us with relevant and insightful comments to improve the manuscript.

After careful consideration, most of these recommendations have been included and highlighted in the revised paper. In particular, we have reworked extensive segments of the Introduction — following Reviewer 1's comments — as well as significantly summarized subsections 3.1, 3.2 and 3.3.3 — as per the combined suggestions of both reviewers. Please see below for a detailed, point-by-point response to each of the comments and suggestions made by the reviewers.

With regards to the dataset associated with this research, we have chosen to make it publicly available in full, as a repository on the Zenodo platform:

- DOI: `10.5281/zenodo.4494912`
- URL: `https://zenodo.org/record/4494912`

Once again, we thank the reviewers for their comments and hope we have managed to sufficiently address their concerns in this revision.

Sincerely,

Manuel Olguín Muñoz
Division of Information Science and
Engineering, EECS
KTH Royal Institute of Technology
Malvinas väg 10, 7th floor
100 44 Stockholm, Sweden
`molguin@kth.se`

Responses to reviewers' comments:

Reviewer 1

Comment 1: Please bring references for (page 2, lines 13–17):

- *One is providing quality of life improvements to the millions of people around the world affected by some form of cognitive decline [. . .]*
- *WCA can, for instance, provide assistance to people recovering from traumatic brain injuries, smoothly guiding them through day-to-day interactions with the world which would otherwise be extremely challenging [. . .]*

Reply: We agree with the reviewer that these claims need further evidence. As such we have decided to rework this segment to highlight the fact that these use cases inspire the work, but they are not everyday reality yet. The paragraphs in question now read:

(Lines 13 *to* 19 *in the revised manuscript.)*

> WCA systems were originally inspired by assistive use cases for people suffering from some form of cognitive decline, either through aging or because of traumatic brain injuries [1, 2]. More recently, they have been applied to a broader range of use cases, including step-by-step guidance on complex assembly tasks [3].
> These systems have also found success as companion tools for specialists and as a means for guiding their training. Non-wearable augmented reality and cognitive assistance systems have already been proven to be valuable tools in the industrial workplace [4, 5]. Detethering this assistance from its current fixed location will surely make it available to many more fields.

Comment 2: *Characterizing the relationships between system responsiveness and user behavior and experience is of paramount importance for the design and evaluation of these applications* (page 2, lines 54–56).
This sentence needs some more clarifications regarding user behaviour and experience: bringing some examples for user behaviour and specifying the experience may help to better understand it. For instance, if the experience is an experience about the use of such applications then it needs to add "use experience" to make it clear.

Reply: We agree with the reviewer, and have thus modified this section of the text to make it more clear by adding some examples:

(Lines 53 to 59 in the revised manuscript.)

> Characterizing the relationships between system responsiveness and user behavior and quality of experience is of paramount importance for the design and evaluation of these applications. It is generally acknowledged, for instance, that a system going from a state of high responsiveness to one of low responsiveness can cause a drop in quality of experience [6]. Furthermore, this could cause users to modify the temporal paramaters of their behavior when interacting with the system, generating a sort of *feedback loop* between user and system. A clear understanding of these relationships would allow, for instance, for the development of strategies for load balancing and optimization for large-scale deployment of WCA.

Comment 3: The research questions cannot be found easily. They should be explained. The aim of the study is mentioned (p3, lines 64–65) i.e. how human behavior changes with system responsiveness. *We aim to tackle this question. . .*, but the research questions need to be clarified too.

Reply: The reviewer is correct in noting that research questions were not explicitly mentioned in the manuscript. We have chosen to address this comment together with Comment 6. Please refer to said comment for details.

Comment 4: *We present in this paper an experimental WCA test-bed of our design and making* (p3, line 70). It seems the sentence is not complete: and making (??)

Reply: We accept that the sentence is unclear and strange. In the revised manuscript it has been changed to:

(Lines 71 to 72 in the revised manuscript.)

> We present in this paper the design and elaboration of an experimental WCA test-bed.

Comment 5: *This test-bed was subsequently employed in a study in which undergraduate students were asked to interact with and follow the instructions given to them by a cognitive assistant* (p3, lines 71–73). Please bring more information about undergraduate students for example in which field, gender. . .

Reply: Yes, for purposes of clarity we have amended the sentence:

(Lines 72 to 74 in the revised manuscript.)

> This test-bed was subsequently employed in a study in which undergraduate students of diverse fields of study, aged between 18 and 25 years old, were asked to interact with and follow the instructions given to them by a cognitive assistant.

We also note that more detailed information about the participants is provided in Section 3.

Comment 6: Research hypotheses should be mentioned and explained in a clear way.

Reply: We agree with the reviewer, and have addressed this comment, together with Comment 3, by reformulating a significant segment of the introduction and explicitly stating the research questions along with the associated hypotheses.

(Lines 8 *to* 121 *in the revised manuscript.)*

> Through this experimental set-up, we intended to answer four core research questions relating to human responses to decreased application responsiveness.
>
> - *Do subjects change the temporal profile of their actions in relation to system latency?*
>
>   In line with previous research in this area, we expected subjects to change their temporal profiles as system responsiveness decreased. The extent or form of these changes were however unknown. We also hypothesized that large enough drops in responsiveness could lead to complete abandonment of the task by subjects.
>
>   Our results show an emergent pacing effect on user actions as system responsiveness is reduced. While it would seem self-evident that users take longer to complete a task while using a system with low responsiveness — as they have to wait longer for new instructions — our study found that user slow-down represents a source of substantial additional delay. To be more precise, the data indicate that users slow down not only because they have to wait for the system to catch up, but that their reactions to new instructions is also delayed. Moreover, this effect scales with the decrease in responsiveness and remains for a while, even after system responsiveness improves.
>
> - *Do subjects show signals of arousal in physiological responses to changes in system latency?*
>
>   We hypothesized subjects would show signs of stress and frustration as system latency increased, due to the added annoyance of dealing with an unresponsive system.
>
>   The results we present, however, seem to refute this hypothesis. We were not able to detect any significant effects on the physiological signals obtained from the biometric sensors as sytem responsiveness was altered.
>
> - *Are responses to delay effects in subjects mediated by cognition and/or emotion?*
>
>   In line with previous items, we expected delay effects in subjects to be mediated primarily by emotion. In particular, we expected emotional effects to be correlated with the strength of the added delay.

The results point in a different direction though, indicating that reduced responsiveness in WCA systems leads to a disruption of participants' cognitive plan for the task and not to an emotional response. This is evidenced by the previously discussed pacing effect and the lack of significant physiological responses.

- *Are these effects mediated by personality indicators in any way?*

  Finally, we hypothesized that the individual trait of *neuroticism* [7] would play a role in mediating these effects, as it has previously been connected to intolerance for time delay [8]. We also expected *focus* and *involvement* [9] to play roles in this.

  The results obtained agree with our hypothesis. We found significant effects of neuroticism on the responses exhibited by subjects, and all three traits were found to play a role through factor analysis.

Comment 7: The introduction part needs to be more elaborated with theoretical frame.

Reply: Agreed. We believe this is partly addressed by the changes made to the introduction according to Comment 3 and Comment 6. Additionally, we have included the following paragraph at the beginning of Section 2:

(Lines 146 *to* 154 *in the revised manuscript.)*

> Our overarching theoretical construct is the idea that people perform temporally sequential tasks in the framework of cognition and emotion. In particular, we view cognition as described by models such as ACT-R [10]; i.e. as a sequence of procedures performed by the subject. Additionally, we hypothesize that individual diferences between subjects moderate responses to delays in the feedback during the execution of a task.
>
> Accordingly, in the following we review background work in the areas of: (Section 2.1) time perception; and (Section 2.2) mechanisms relating human performance to delay.

Comment 8: As the authors wrote a *third potential explanation of delay effects* (p5, line 167). Similar types of writing for all three explanation may help readers grasp the messages easier: thus, I suggest that the authors bring at the beginning of previous relevant phrases "first explanation" and "second explanation" too.

Reply: Yes. The explanations in question have been modified in the revised manuscript:

(Lines 191 *to* 192 *in the revised manuscript.)*

> A first possible explanation comes from research on cognitive and motor planning. Delay may move users from relatively automatic to more attention-demanding processing. [...]

(Lines 205 *to* 206 *in the revised manuscript.)*

> A second, alternative explanation of delay effects appeals to emotional systems rather than cognitive processes. [...]

(Lines 208 *to* 210 *in the revised manuscript.)*

> Finally, a third potential explanation of delay effects is what has been called "ego depletion", the notion that expending effort on self-control eliminates resources needed for further effort. [...]

Comment 9: Key characteristics of the sample (Section 3) should be clarified.

Reply: Agreed. We assume here that by *sample* the reviewer refers to the *participant population*. We have amended the text to specify that our participants correspond to undergraduate volunteers enrolled in an introductory-level psychology course at CMU:

(Lines 240 *to* 250 *in the revised manuscript.)*

> This study was conducted with the approval of the Carnegie Mellon University Institutional Review Board under record number `STUDY2019_00000247`. Written consent was obtained.
>
> Subjects were recruited from a pool of undergraduate students at Carnegie Mellon University. Students enrolled in an introductory-level psychology course fulfill a research requirement as part of the plan of study. This requirement may be fulfilled either through the elaboration of a written essay or by volunteering as a participant in a small number of research experiments.
>
> No particular exclusion criteria were applied, and as specified by our approved data-collection protocol, no gender- or sex-related statistics were collected. In total, 40 participants were recruited, all of them of college student age (18 to 25 years old).

Comment 10: Sections 3.1, 3.2 need to be summarized. This part regarding definitions are too long and need to be shortened.

Reply: Yes, we agree with the reviewer, and have edited sections 3.1 and 3.2 to be more concise. As the changes are too extensive to fully detail here, please refer to the revised manuscript, specifically lines 250 to 324.

Comment 11: Subsection 3.3.3 also need to be shortened and summarized in a clearer way. This part is very long too. For instance, EEG is well known, it doesn't need to be defined very much.

Reply: We agree. Subsection 3.3.3 has been shortened by summarizing descriptions and trimming away unnecessary details. It now reads as follows:

(Lines 357 to 385 in the revised manuscript.)

The participants wore devices to acquire four physiological measures: (1) galvanic skin response (*GSR*), (2) accelerometer data from the dominant wrist, (3) brain activity in the form of electroencephalography (*EEG*), and (4) heart rate.

These metrics have been used as indicators of stress and cognitive load by an ample body of previous research [11–13]. GSR (also known as electrodermal activity) can be interpreted as an indicator of physiological arousal and has long been a widely used metric in studies seeking to characterize mental workload [11–16]. EEG on the other hand has previously been used to measure cognitive load in the context of human-computer interactions [17–19].

Wrist acceleration, GSR and heart rate data were obtained using the Empatica E4 [20] biosensing wristband. Accelerometer data was sampled at 32 Hz, GSR was sampled at 4 Hz, and instantaneous heart rate was calculated from a *blood volume pulse* (BVP) signal sampled at 64 Hz. Participants were asked to wear the device for approximately 10–15 minutes before starting the experiment, in order to allow the sensors to reach a stable equilibrium and establish a baseline for the signals.

The E4 wristband was chosen due to its small, non-invasive, and wireless form factor (samples were streamed to the system over Bluetooth LE) and for the fact that its use in research has been experimentally validated in previous studies [21, 22]. The E4 also includes a skin temperature thermometer, however the measure was not used for the present study.

For the EEG data we employed the OpenBCI EEG Headband Kit[23] consisting of a number of dry electrodes fastened to a Velcro headband. It provides a quick and non-invasive way of obtaining EEG signals from participants. Electrodes were placed according to the *10–20 Electrode System* [24] on the *Fp1* and *Fp2* points, in order to capture brain activity in the frontal lobe, with ground and reference electrodes on the right and left earlobes, respectively. The signals were sampled at 200 Hz and postprocessed to (1) remove frequencies outside the 0.1 Hz to 40 Hz range, and (2) smooth out noise (using the technique proposed by Agarwal et al. [25]).

Comment 12: [The Discussion] part is very short in comparison to other parts of this study. The reflection of the authors of the current paper on their results is missing (the reflection of authors regarding why some results were significant while other not are important). Thus the authors should elaborate the Discussion part as well.

Reply: We partially agree with the reviewer. We believe that this concern of the reviewer was addressed in the original discussion section (paragraph beginning with line 597 in the old manuscript), but apparently not with sufficient clarity. We pointed out that the failure of the physiological measures to show effects of delay indicates that resource depletion and emotionally induced arousal are not impacted. We concluded, "Thus, contrary to our preliminary postulations, behavioral effects seem to arise from impaired cognitive control mechanisms, and not from emotion or resource depletion."
In response to the review, we have now expanded this paragraph to specifically point to the statistical outcomes. That is, we now say:

(Lines 596 *to* 602 *in the revised manuscript.)*

> However, the present data provide relatively little support for these alternatives, in that the predicted measures did not produce the expected statistical trends. Specifically, physiological measures of GSR and HR failed to show evidence of differential arousal under long vs. short delays, and speed-induced errors and non-completions predicted by resource depletion were not observed. The acceleration data further did not indicate that extended delay significantly increases erratic movement.

Reviewer 2

Comment 1: The explanations have been used too much in somewhere. For example, it is not necessary to explain the ANOVA test. This test is well known.

Reply: We agree with the reviewer. The explanation of the ANOVA test has been removed, and the paragraph has been reworked with a new reference:

(Lines 411 *to* 413 *in the revised manuscript.)*

> We confirmed this effect through an *analysis of variance* (ANOVA [26]) with factors of block length and delay, and found significant main effects of both factors and the interaction, shown in Table 2.

We also understand this remark as relating to the same issues pointed out by *Reviewer 1* in Comment 10 and Comment 11. As such, we refer to our responses to these comments.

Comment 2: Use more resolution for figures.

Reply: Yes, this has been addressed in the revised manuscript.

Comment 3: Some figures can be inserted in a figure as subplots and or subpanels.

Reply: Agreed. This has been addressed in the revised manuscript. In particular, figure 4 has been reorganized and merged with figure 5 (in the old manuscript).

Comment 4: In line 286 at the end of line the "A Latin" is written, what is that?

Reply: This is an artifact of the line-wrapping of the document. The segment of text actually reads "A *Latin square*-type design [...]", but LaTeXdecided to automatically wrap the line right after *Latin*. This has been fixed in the revised manuscript.

References

[1] K. Ha, Z. Chen, W. Hu, W. Richter, P. Pillai, and M. Satyanarayanan, "Towards wearable cognitive assistance," in *Proceedings of the 12th Annual International Conference on Mobile Systems, Applications, and Services*, ser. MobiSys '14, Bretton Woods, New Hampshire, USA: ACM, 2014, pp. 68–81, ISBN: 978-1-4503-2793-0. DOI: 10.1145/2594368.2594383. [Online]. Available: http://doi.acm.org/10.1145/2594368.2594383.

[2] M. Satyanarayanan and N. Davies, "Augmenting Cognition Through Edge Computing," *Computer*, vol. 52, no. 7, pp. 37–46, 2019. DOI: 10.1109/MC.2019.2911878.

[3] Z. Chen, W. Hu, J. Wang, S. Zhao, B. Amos, G. Wu, K. Ha, K. Elgazzar, P. Pillai, R. Klatzky, D. Siewiorek, and M. Satyanarayanan, "An empirical study of latency in an emerging class of edge computing applications for wearable cognitive assistance," in *Proceedings of the Second ACM/IEEE Symposium on Edge Computing*, ser. SEC '17, San Jose, California: ACM, 2017, 14:1–14:14, ISBN: 978-1-4503-5087-7. DOI: 10.1145/3132211.3134458. [Online]. Available: http://doi.acm.org/10.1145/3132211.3134458.

[4] M. Funk and A. Schmidt, "Cognitive assistance in the workplace," *IEEE Pervasive Computing*, vol. 14, no. 3, pp. 53–55, 2015.

[5] D. Gorecky, S. F. Worgan, and G. Meixner, "COGNITO: A Cognitive Assistance and Training System for Manual Tasks in Industry," in *Proceedings of the 29th Annual European Conference on Cognitive Ergonomics*, ser. ECCE '11, Rostock, Germany: Association for Computing Machinery, 2011, pp. 53–6, ISBN: 9781450310291. DOI: 10.1145/2074712.2074723. [Online]. Available: https://doi.org/10.1145/2074712.2074723.

[6] J. Dabrowski and E. V. Munson, "40 years of Searching for the Best Computer System Response Time," *Interact. Comput.*, vol. 23, no. 5, 555–564, Sep. 2011, ISSN: 0953-5438. DOI: 10.1016/j.intcom.2011.05.008. [Online]. Available: https://doi.org/10.1016/j.intcom.2011.05.008.

[7] O. P. John, S. Srivastava, *et al.*, "The big five trait taxonomy: History, measurement, and theoretical perspectives," *Handbook of personality: Theory and research*, vol. 2, no. 1999, pp. 102–138, 1999.

[8] J. B. Hirsh, D. Morisano, and J. B. Peterson, "Delay discounting: Interactions between personality and cognitive ability," *Journal of research in personality*, vol. 42, no. 6, pp. 1646–1650, 2008.

[9] B. G. Witmer and M. J. Singer, "Measuring presence in virtual environments: A presence questionnaire," *Presence*, vol. 7, no. 3, pp. 225–240, 1998.

[10] D. M. Neves and J. R. Anderson, "Knowledge compilation: Mechanisms for the," *Cognitive skills and their acquisition*, vol. 6, p. 57, 1981.

[11] A. Khawaji, J. Zhou, F. Chen, and N. Marcus, "Using Galvanic Skin Response (GSR) to Measure Trust and Cognitive Load in the Text-Chat Environment," in *Proceedings of the 33rd Annual ACM Conference Extended Abstracts on Human Factors in Computing Systems*, ser. CHI EA '15, Seoul, Republic of Korea: Association for Computing Machinery, 2015, 1989–1994, ISBN: 9781450331463. DOI: 10.1145/2702613.2732766. [Online]. Available: https://doi-org.focus.lib.kth.se/10.1145/2702613.2732766.

[12] K. Kuikkaniemi, T. Laitinen, M. Turpeinen, T. Saari, I. Kosunen, and N. Ravaja, "The influence of implicit and explicit biofeedback in first-person shooter games," in *Proceedings of the SIGCHI Conference on Human Factors*

in Computing Systems, ser. CHI '10, Atlanta, Georgia, USA: Association for Computing Machinery, 2010, 859–868, ISBN: 9781605589299. DOI: 10.1145/1753326.1753453. [Online]. Available: https://doi-org.focus.lib.kth.se/10.1145/1753326.1753453.

[13] E. T. Solovey, M. Zec, E. A. Garcia Perez, B. Reimer, and B. Mehler, "Classifying driver workload using physiological and driving performance data: Two field studies," in *Proceedings of the SIGCHI Conference on Human Factors in Computing Systems*, ser. CHI '14, Toronto, Ontario, Canada: Association for Computing Machinery, 2014, 4057–4066, ISBN: 9781450324731. DOI: 10.1145/2556288.2557068. [Online]. Available: https://doi-org.focus.lib.kth.se/10.1145/2556288.2557068.

[14] F. Peterson and C. G. Jung, "Psycho-physical investigations with the galvanometer and pneumograph in normal and insane individuals," *Brain*, vol. 30, pp. 153–218, 1907.

[15] J. A. Healey and R. W. Picard, "Detecting stress during real-world driving tasks using physiological sensors," *IEEE Transactions on Intelligent Transportation Systems*, vol. 6, no. 2, pp. 156–166, Jun. 2005. DOI: 10.1109/tits.2005.848368. [Online]. Available: https://doi.org/10.1109/tits.2005.848368.

[16] J. Son, B. Reimer, B. Mehler, A. E. Pohlmeyer, K. M. Godfrey, J. Orszulak, J. Long, M. H. Kim, Y. T. Lee, and J. F. Coughlin, "Age and cross-cultural comparison of drivers' cognitive workload and performance in simulated urban driving," *International Journal of Automotive Technology*, vol. 11, no. 4, pp. 533–539, Jul. 2010. DOI: 10.1007/s12239-010-0065-6. [Online]. Available: https://doi.org/10.1007/s12239-010-0065-6.

[17] P. Antonenko, F. Paas, R. Grabner, and T. van Gog, "Using electroencephalography to measure cognitive load," *Educational Psychology Review*, vol. 22, no. 4, pp. 425–438, Apr. 2010. DOI: 10.1007/s10648-010-9130-y. [Online]. Available: https://doi.org/10.1007/s10648-010-9130-y.

[18] D. Grimes, D. S. Tan, S. E. Hudson, P. Shenoy, and R. P. N. Rao, "Feasibility and pragmatics of classifying working memory load with an electroencephalograph," in *Proceeding of the twenty-sixth annual CHI conference on Human factors in computing systems - CHI '08*, ACM Press, 2008. DOI: 10.1145/1357054.1357187. [Online]. Available: https://doi.org/10.1145/1357054.1357187.

[19] N. Kumar and J. Kumar, "Measurement of cognitive load in HCI systems using EEG power spectrum: An experimental study," *Procedia Computer Science*, vol. 84, pp. 70–78, 2016.

[20] (2020). "Empatica E4." [Online; accessed 24 Mar 2020], [Online]. Available: https://www.empatica.com/research/e4/.

[21] M. Ragot, N. Martin, S. Em, N. Pallamin, and J. M. Diverrez, "Emotion recognition using physiological signals: Laboratory vs. wearable sensors," in *International Conference on Applied Human Factors and Ergonomics*, Springer, 2017, pp. 15–22.

[22] C. McCarthy, N. Pradhan, C. Redpath, and A. Adler, "Validation of the Empatica E4 wristband," in *2016 IEEE EMBS International Student Conference (ISC)*, IEEE, 2016, pp. 1–4.

[23] (2020). "OpenBCI EEG Headband Kit." [Online; accessed 20 Mar 2020], [Online]. Available: https://shop.openbci.com/products/openbci-eeg-headband-kit?variant=8120393760782.

[24] "The ten twenty electrode system: International federation of societies for electroen-

cephalography and clinical neurophysiology," *American Journal of EEG Technology*, vol. 1, no. 1, pp. 13–19, 1961. DOI: 10.1080/00029238.1961.11080571. eprint: https://doi.org/10.1080/00029238.1961.11080571. [Online]. Available: https://doi.org/10.1080/00029238.1961.11080571.

[25] S. Agarwal, A. Rani, V. Singh, and A. P. Mittal, "EEG signal enhancement using cascaded S-Golay filter," *Biomedical Signal Processing and Control*, vol. 36, pp. 194–204, 2017.

[26] Y. Fujikoshi, "Two-way ANOVA models with unbalanced data," *Discrete Mathematics*, vol. 116, no. 1, pp. 315 –334, 1993, ISSN: 0012-365X. DOI: https://doi.org/10.1016/0012-365X(93)90410-U. [Online]. Available: http://www.sciencedirect.com/science/article/pii/0012365X9390410U.
